# Loss of TGF-β or Wnt5a results in an increase in Wnt/β-catenin activity and redirects mammary tumour phenotype

**DOI:** 10.1186/bcr2244

**Published:** 2009-04-03

**Authors:** Kevin Roarty, Sarah E Baxley, Michael R Crowley, Andra R Frost, Rosa Serra

**Affiliations:** 1 Department of Cell Biology, University of Alabama at Birmingham, 1918 University Boulevard, MCLM 660, Birmingham, AL 35294-0005, USA; 2 Department of Genetics, University of Alabama at Birmingham, 720 20th Street South, Birmingham, Alabama 35294, USA; 3 Department of Pathology, Division of Anatomic Pathology, University of Alabama at Birmingham, West Pavilion P220, 619 South 19th Street, Birmingham, AL 35233-7331, USA

## Abstract

**Introduction:**

The tumour-suppressive effects of transforming growth factor-beta (TGF-β) are well documented; however, the mechanistic basis of these effects is not fully understood. Previously, we showed that a non-canonical member of the Wingless-related protein family, Wnt5a, is required for TGF-β-mediated effects on mammary development. Several lines of evidence support the hypothesis that Wnt5a acts as a tumour suppressor. In addition, it has been shown that Wnt5a can antagonise canonical Wnt/β-catenin signalling in various cell types. Here we test the hypothesis that TGF-β and Wnt5a can antagonise Wnt/β-catenin signalling and redirect mammary tumour phenotype. The results provide a new mechanism for the tumour-suppressive effects of TGF-β.

**Methods:**

Wnt/β-catenin signalling was measured in tumours with altered TGF-β (dominant-negative TGF-β type II receptor, DNIIR) or Wnt5a (*Wnt5a*^-/-^) signalling as the accumulation of nuclear β-catenin using both confocal microscopy and cell fractionation. RT-PCR was used to measure the expression of Wnt/β-catenin target genes. Sca1 expression was determined by western blot and keratin (K) 6- and K14-positive populations were determined by immunohistochemistry.

**Results:**

Loss of TGF-β or Wnt5a signalling resulted in stabilisation of nuclear β-catenin and expression of Wnt/β-catenin target genes suggesting that TGF-β and Wnt5a act to inhibit Wnt/β-catenin signalling in mammary epithelium. Increased expression of Sca-1 was observed in developing DNIIR and *Wnt5a*-/- mammary glands. DNIIR and Wnt5a-/- tumours demonstrated an expanded population of K6- and K14-expressing cells typically seen in Wnt/β-catenin-induced tumours.

**Conclusions:**

The key findings here are that: TGF-β and Wnt5a regulate Wnt/β-catenin activity; and loss of TGF-β and Wnt5a redirect the phenotype of tumours so that they resemble tumours induced by activation of Wnt/β-catenin. The findings suggest a new mechanism for the tumour-suppressive effects of TGF-β.

## Introduction

Transforming growth factor-β (TGF-β) is responsible for the maintenance of homeostasis in many epithelial tissues that exhibit a high rate of cell turnover. Consequently, disruption of TGF-β signalling is attributed to the development of human carcinomas including colon, gastric and breast cancers [1,2]. Ligand binding to the type II TGF-β receptor (TGF-βRII) recruits and activates the type I TGF-β receptor (TGF-βRI) via serine/threonine phosphorylation, thereby activating downstream Smad signal transducers. Although mutations in genes encoding proteins in the TGF-β signalling pathway are uncommon in human breast cancers, loss or diminished expression of TGF-βRII confers a 3.4-fold greater risk of developing invasive breast carcinoma in a subset of patients [3] and is associated with higher histological grade in human breast *in situ *and invasive carcinomas [4]. Moreover, using both gain- and loss-of-function approaches, a variety of transgenic mouse models support a tumour-suppressive role for TGF-β in mammary cancers (reviewed in [2]). For example, overexpression of TGF-β1 in the mammary epithelium via MMTV-TGF-β1 or WAP-TGF-β1 inhibits the formation of TGF-α and 7, 12-dimethybenz(a)anthracene (DMBA)-induced tumours. In contrast, interruption of endogenous TGF-β signalling via overexpression of a dominant-negative TGF-βRII mutant (DNIIR) or Cre/Lox deletion of Tgfbr2 within the mammary epithelium increases tumour incidence. Recently, we identified a non-canonical member of the Wingless-related protein family, Wnt5a, as a downstream target for TGF-β in mammary gland development [5].

Like the TGF-β family of polypeptides, Wnt proteins make up a large family of essential signalling molecules that mediate a range of cellular behaviours including cell fate specification, cell polarity and cell migration [6]. Moreover, mutations or alterations in the Wnt pathway contribute to a variety of human cancers, the majority of which exhibit deregulated intracellular β-catenin levels and, thus, increased β-catenin activity. Evidence suggests the Wnt/β-catenin pathway preferentially induces mammary cancers from progenitor cells, with tumours exhibiting expansion of undifferentiated keratin (K) 6 and Sca-1-positive populations, along with both luminal and uncharacteristic myoepithelial lineages throughout the tumours. Although most studies have focused on the Wnt/β-catenin pathway, other Wnt signalling pathways exist that signal independently of β-catenin, so-called non-canonical pathways. Interestingly, studies suggest that Wnt5a, a non-canonical signalling Wnt, can antagonise the Wnt/β-catenin pathway; however, the mechanism and biological consequences of this antagonism vary by tissue type [7-10].

Several lines of evidence support the hypothesis that Wnt5a acts as a tumour suppressor. Overexpression of Wnt5a is able to partially revert the transformed phenotype of C57MG cells *in vitro *after ectopic expression of Wnt1, while anti-sense suppression of Wnt5a expression causes a similar cell morphology exhibited by Wnt1-transformed C57MG cells [11]. Loss of Wnt-5a protein expression in invasive ductal carcinomas also correlates with an increased risk of early relapse and mortality for patients [12,13]. Additionally, Wnt5a mRNA levels seem to decline with more advanced stages of breast disease [13]. In light of solid credentials in favour of Wnt5a as a tumour suppressor, other studies present evidence for an oncogenic role for Wnt5a [14,15]. It is likely that the tumour-suppressive and oncogenic effects of Wnt5a are highly dependent on the cell type and context in which the signalling occurs; however, these discrepancies clearly emphasise the need for further investigation.

Here, we show that dominant-negative interference of TGF-β signalling in tumours results in reduced Wnt5a expression. Next, we showed that expression of DNIIR or loss of Wnt5a in mammary tissue enhances Wnt/β-catenin activity. In addition, loss of TGF-β or Wnt5a signalling results in increased Sca1 expression and redirection of tumour phenotype to that consistent with excess Wnt/β-catenin signalling, including an increase in K6- and K14-positive cell populations. We propose that Wnt5a acts to mediate some of the tumour-suppressive effects of TGF-β by antagonising the Wnt/β-catenin pathway.

## Materials and methods

### Mice

All mice in this study were maintained under the guidelines of the Institutional Animal Care and Use Committee of the University of Alabama at Birmingham. DNIIR mice were previously described [16,17]. *Wnt5a *disrupted mice (*Wnt5a*^tm1/Amc^) were acquired from Jackson Laboratories (Bar Harbor, Maine, USA) and backcrossed at least five times into a C57Bl/6 background before generating *Wnt5a*^-/- ^embryos. ICR/SCID mice were obtained from Taconic Farms (Hudson, NY, USA). MMTV-PyVmT strains were acquired from Jackson Laboratories and were also backcrossed to a C57BL/6J background. MMTV-neu mice (FVB) were crossed with hemizygous DNIIR (C57BL/6) mice to obtain F1 generation MMTV-neu and MMTV-neu:DNIIR groups. MMTV-PyVmT male mice were crossed to hemizygous DNIIR females to obtain MMTV-PyVmT and MMTV-PyVmT:DNIIR groups.

### Generation of Wnt5a-/- and PyVmT:Wnt5a-/- epithelium

Rescue of Wnt5a-/- epithelium has been previously described [5]. PyVmT:Wnt5a-/- epithelium was generated by crossing MMTV-PyVmT males with Wnt5a+/- females to acquire MMTV- PyVmT:Wnt5a+/- males that were then bred with Wnt5a+/- females to yield MMTV-PyVmT and PyVmT:Wnt5a-/- embryos. Mammary anlagen were then microdissected from e18.5 day embryos. The buds were pooled and cultured overnight to form one transplantable unit that was then implanted the next day into the cleared fat pads of three-week-old ICR/SCID hosts [18]. During culture of the mammary anlagen overnight, Wnt5a-/- embryos were screened for the PyVmT transgene. Rescued epithelium was expanded within the fat pad of ICR/SCID hosts for one month before being removed and transplanted into cleared host fat pads for experiments.

### Generation of organoid preparations for injection into fat pad

For PyVmT:DNIIR studies, organoid preparations were prepared from control and experimental groups at three weeks of age to obtain a homogeneous population of cells for injection into the cleared mammary fat pads of ICR/SCID hosts. For PyVmT:Wnt5a-/- studies, organoid preparations were prepared from rescued mammary epithelium that was expanded within the fat pad. Glands from MMTV-PyVmT and MMTV-PyVmT:DNIIR groups, in addition to rescued MMTV-PyVmT and MMTV-PyVmT:Wnt5a-/- tissue, were minced and enzymatically dissociated in 10 ml digestion media/1 g of tissue (digestion medium: 1 mg/ml type I collagenase and 0.1 mg/ml pronase in Dulbecco's modified eagle's medium (DMEM)/F12) for one hour at 37°C at 125 rpm on a rotary shaker. Samples were pipetted every 20 minutes to expedite dissociation.

Organoids were obtained by differential centrifugation, washed three times in 1 × PBS, and resuspended in DMEM/F12. A 50 μl volume of 50 organoids in 1 × Hanks' balanced salt solution (HBSS) was injected into each fat pad. Isolation of organoid preparations was consistent between control and experimental groups, and although organoid sizes vary, this variability appeared consistent by microscopic visualisation of the organoids before injection. Successful delivery of organoid suspensions was confirmed by visible engorgement of the fat pad upon injection [19].

### Tissue processing and immunohistochemistry

Tissues were fixed in 4% paraformaldehyde overnight at 4°C and processed to paraffin block after graded ethanol dehydration steps and clearance in xylene. Sodium citrate (10 mM, pH 6) antigen retrieval was used in all immunohistochemistry and immunofluorescence procedures. Immunohistochemistry was carried out using the Vector ABC kit (Vector Laboratories, Burlingame, CA, USA) at the manufacturer's recommendations. For immunofluorescence, sections were probed with biotinylated secondary antibodies (1:250; Vector Laboratories) followed by Cy-3 conjugated streptavidin (1:1000; Zymed Laboratories, San Francisco, CA, USA; 43-4315). BrdU and TUNEL (terminal deoxynucleotidyl transferase dUTP nick end labeling) staining have been described previously [5]. Primary antibodies included anti-β-catenin (1:500; Cell Signaling Technologies, Danvers, MA, USA; #9562), anti-keratin-6 (1:500; Covance, Princeton, NJ, USA), anti-keratin-14 (1:1000; Covance; PRB-155P) and anti-CD31 (1:200; NeoMarkers, Freemont, CA, USA; RB-10333-P0).

Images of the H&E-stained tumours, as well as all of the Ki67, Brdu, CD31, K14 and K6 stained tissues, were taken using the 40× objective on an Olympus BX50 microscope (Olympus, Center Valley, PA, USA), a magnafire camera (Olympus) and PictureFrame software (Olympus). All images were opened, colours merged and adjusted for sharpness in Adobe Photoshop (Adobe, San Jose, CA, USA). Confocal image were taken using the 100× oil objective on a Nikon Eclipse TE2000-U microscope with a Perkin Elmer Ultra View spinning disc set-up and an EMCCD C9100-50 camera using Ultraview ERS acquisition software (Ultraview, Waltham, MA, USA). Figures were composed using Adobe Photoshop.

### Isolation and treatment of mammary epithelial cells with recombinant Wnt proteins

Adult virgin Balb/c mice (8 to 10 weeks of age) were the source of primary mammary epithelial cells. Cell fractions were isolated by enzymatic dissociation and Percoll gradient density centrifugation as previously described [20]. Fibroblasts were plated in DMEM/F12 supplemented with 5% fetal bovine serum (FBS), 100 μg/ml penicillin and 50 μg/ml streptomycin, were allowed to attach for two hours, were rinsed with 1 × PBS and were refreshed with growth medium. The epithelial cells were plated 1 × 10^5 ^cells/cm^2 ^in DMEM/F12 supplemented with 5% FBS, 20 ng/ml epidermal growth factor (EGF), 0.5 μg/ml hydrocortisone, 100 ng/ml cholera toxin, 10 μg/ml insulin, 100 μg/ml penicillin and 5 μg/ml streptomycin (Clonetech, Mountain View, CA, USA). Cultures were allowed to grow until 70% confluent, and then serum starved overnight in basal medium (DMEM/F12 supplemented with 0.1 mM non-essential amino acids, 0.1 μg/ml insulin, 1 mg/ml fatty acid-free BSA (fraction V), 100 μg/ml penicillin and 50 μg/ml streptomycin) (Clonetech) prior to treatment with recombinant Wnt proteins. rWnt5a and rWnt3a were purchased from R&D systems (Minneapolis, MN, USA) (rmWnt5a cat# 645-WN-010 and rmWnt3a cat# 1324-WN-002).

### Mammary gland and tumour subcellular fractionation and western blotting

Single cell suspensions of mammary glands and tumours were obtained before subcellular fractionation procedures. Glands and tumours were minced and enzymatically dissociated in 10 ml digestion media/1 g tissue (see *organoid preparation *section) to arrive at an organoid preparation. The pelleted organoids were resuspended in 2 ml trypsin-EDTA (1 mM EDTA-4NA, 2.5 g/L porcine trypsin, in HBSS, Ca^++ ^Mg^++ ^free), and pipetted up and down for one minute before adding 8 ml modified HBSS (with 10 mM 4-(2-hydroxyethyl)-1-piperazineethanesulfonic acid (HEPES)) supplemented with 2% FBS. This suspension was centrifuged at 350 × g for five minutes. The organoids were further dissociated in a Dispase/DnaseI solution (2 ml of 5 mg/ml Dispase + 200 μl 1 mg/ml DnaseI; Invitrogen, Carlsbad, CA, USA), resuspended in modified HBSS, filtered through a 40 μm filter, and washed three times in 1 × PBS. After washing, the pellet of single mammary and tumour cells was fractionated into cytosolic and nuclear compartments, as previously described [21]. Protein lysate from cell fractions was resolved on an 8% SDS-PAGE gel, transferred to polyvinylidene fluoride membrane and probed with the indicated antibodies anti-β-catenin (1:1000, Cell Signaling Technologies), anti-Sca1, (1:1000, R&D systems; #AF1226), anti-Creb (1:1000; AbCam, Cambridge, MA, USA), anti-β-tubulin (1:1000; Santa Cruz, Santa Cruz, CA, USA) according to standard protocols.

### RNA Isolation and semi-quantitative RT-PCR

RNA isolation was performed using Trizol^® ^reagent according to the manufacturer's instructions. Relative levels of mRNAs were determined using semi-quantitative RT-PCR. cDNA was synthesised from 5 μg total RNA using random primers. cDNA was then amplified using specific primers as follows: 18Sfwd 5'-ACGGAAGGGCACCACCAGG-3', 18Srev 5'-CACCAACTAAGAACGGCCATGC-3', tcf-4fwd 5'-ATGGCCATCACAGCAGCGAC-3', tcf-4rev 5'-CTGTCTGTTCCGTTGGCAGGG-3', c-mycfwd 5'-CGATGCCCCTCAACGTGAAC-3', c-mycrev 5'-CAGGCTGGTGCTGTCTTTGC-3', cyclin D1 F 5'-GTCTGTGAGGAGCAGAAGTGC-3', cyclinD1 R 5'-GGCCGGATAGAGTTGTCTGTG-3', axin2F 5'-GAGTAGCGCCGTGTTAGTGACT-3', axin2R 5'-CCAGGAAAGTCCGGAAGAGGTATG-3', Sca1fwd 5'-GCCCATCAATTACCTGCCC-3', Sca1rev 5'-CCTGGCAACAGGAAGTCTTG-3'. Amplification of cDNA was performed over varying cycles to arrive at product formation in the linear range.

### Statistical methods

The length and width of the tumours were measured with a caliper. Tumour volume was calculated by the formula: volume = (width^2 ^× length)/2. The analysis of subcutaneous MMTV-neu tumour volume was performed using a repeated measures model with PROC MIXED (SAS Version 9.1, Cary, NC, USA). The *a priori *planned comparisons of specific differences in predicted treatment means at 63 days were computed by t-statistics (Student's t-test). For all analyses a two-sided p value of less than 0.05 was deemed statistically significant.

For other tumour volume measurements, proliferation and CD31 vessel density, mean values were calculated then statistical significance was evaluated based on t-statistics and plots were generated using GraphPad Prism version 4.00 for Windows (GraphPad Software, San Diego, CA, USA).

## Results

### Interruption of TGF-β signalling enhances tumour growth accompanied by diminished Wnt5a protein levels

Previously, we showed that expression of Wnt5a is positively regulated by TGF-β, mimicks the inhibitory effects of TGF-β during mammary development and is required for TGF-β-mediated inhibition of branching *in vivo *and *in vitro *[5]. To determine the effects of alterations in TGF-β signalling (DNIIR) on Wnt5a expression during mammary tumour progression we used the previously characterised MMTV-neu [22] and MMTV-PyVmT [23] models of mouse mammary tumourigenesis. Dominant-negative interference of TGF-β signalling was achieved by crossing MMTV-neu or MMTV-PyVmT mice with the MT-DNIIR model that has been previously described [17]. MMTV-neu;DNIIR tumours were spontaneous tumours generated in the F1 generation of MMTV-neu and DNIIR crosses. In the MMTV-PyVmT study, tumours were generated by transplanting organoids isolated from MMTV-PyVmT or MMTV-PyVmT;DNIIR mammary glands into Scid hosts. Time of tumour detection was denoted by the presence of a palpable 2 × 2 mm mass within the fat pad. For the MMTVneu study, mice were sacrificed when the tumour volume reached 2 cm or if the mice became moribund. The study ended at 14 months and all remaining mice were sacrificed. MMTV-PyVmT tumours arise much more quickly, so all the mice on the MMTV-PyVmT;DNIIR study were sacrificed at 10 weeks (70 days). All tumours generated here were classified as invasive adenocarcinomas.

**Figure 1:**
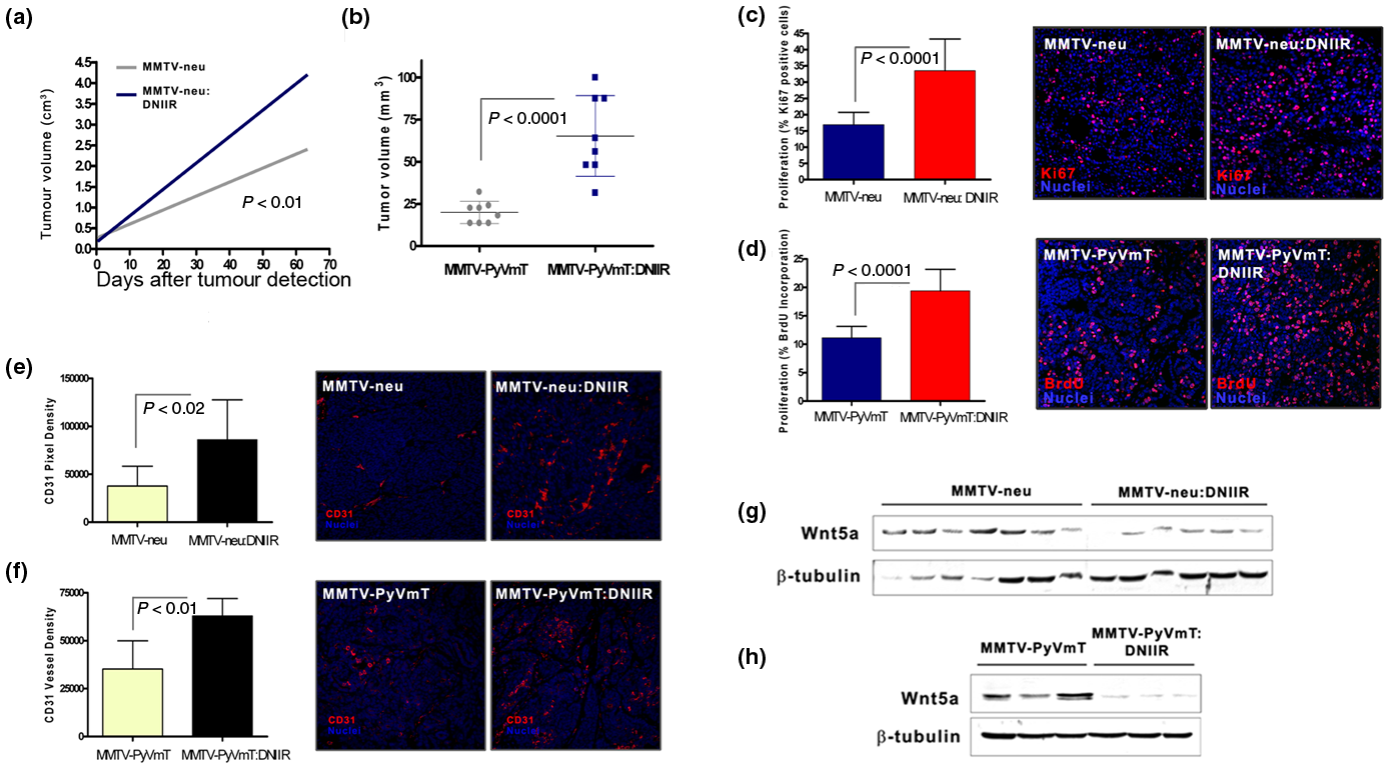
Wnt5a protein levels are reduced in DNIIR tumours. **(a) **Repeated measures modelling of tumour volume over time in the MMTV-neu model shows statistically significant differences at 63 days in mean tumour volume between control and DNIIR groups (*P *< 0.01; control = 2.57 cm^3 ^± 0.53 cm^3^, n = 12; DNIIR = 4.21 cm^3 ^± 0.17 cm^3^, n = 33). **(b) **In the MMTV-PyVmT model, a graphical plot of mean tumour volumes at the time of sacrifice reveals significantly larger tumours in the DNIIR group versus controls (*P *< 0.0001; control = 20 mm^3^, n = 8; DNIIR = 65 mm^3^, n = 8). **(c) **Ki67 staining in the MMTVneu model shows increased levels of proliferation in the DNIIR primary tumours compared with controls (*P *< 0.0001; six tumours per group, five fields per tumour were counted). **(d) **BrdU incorporation in tumours of the MMTV-PyVmT study illustrates increased proliferation in DNIIR tumours (*P *< 0.0001; eight tumours per group, three fields per tumour). **(e, f) **Vascular density, measured by CD31 pixel density, was greater in DNIIR groups within both (e) MMTV-neu and (f) MMTV-PyVmT studies (MMTV-neu = *P *<0.02, five tumours per group, three fields per tumour; MMTV-PyVmT = *P *< 0.01, four tumours per group, three fields per tumour). **(g, h) **Western blotting for Wnt5a protein in lysates from several separate (g) MMTV-neu and (h) MMTV-PyVmT tumours with or without DNIIR, indicating a decrease in overall levels of Wnt5a protein in primary tumours with alterations in TGF-β signalling. β-Tubulin is used as a loading control. DNIIR = dominant-negative TGF-β type II receptor; MMTV = mouse mammary tumour virus promoter/enhancer; PyVmT = polyoma virus middle T antigen; TGF-β = τransforming growth factor-beta; Wnt = Wingless-related protein family.

Repeated measures modelling was used to extrapolate tumour volume at 63 days post-detection for MMTV-neu and MMTV-neu;DNIIR tumours. The results indicated significantly faster growth and larger tumours in the MMTV-neu:DNIIR group compared with the control MMTV-neu (Figure 1a). Likewise, MMTV-PyVmT:DNIIR tumours were larger at the time of sacrifice (70 days) compared with control MMTV-PyVmT tumours (Figure 1b). In both models, overexpression of DNIIR increased proliferation rates in the primary tumours, illustrated by Ki67 staining or BrdU incorporation (Figures 1c, d). No statistical differences in apoptosis were observed in tumours harboring intact versus altered TGF-β signalling (data not shown).

We also identified differences in microvessel density between control and DNIIR groups. In both MMTV-neu and MMTV-PyVmT models, overexpression of DNIIR resulted in an increase in the extent of vasculature throughout the primary tumours, depicted by CD31 immunostaining (Figures 1e, f).

By western blotting, Wnt5a protein levels were reduced in tumours with altered TGF-β responsiveness in both the MMTV-neu (Figure 1g) and MMTV PyVmT (Figure 1h) models suggesting that, in addition to regulating Wnt5a in normal mammary development, TGF-β can act to maintain Wnt5a expression during tumour formation.

### Absence of Wnt5a promotes tumour growth and accumulation of β-catenin

**Figure 2:**
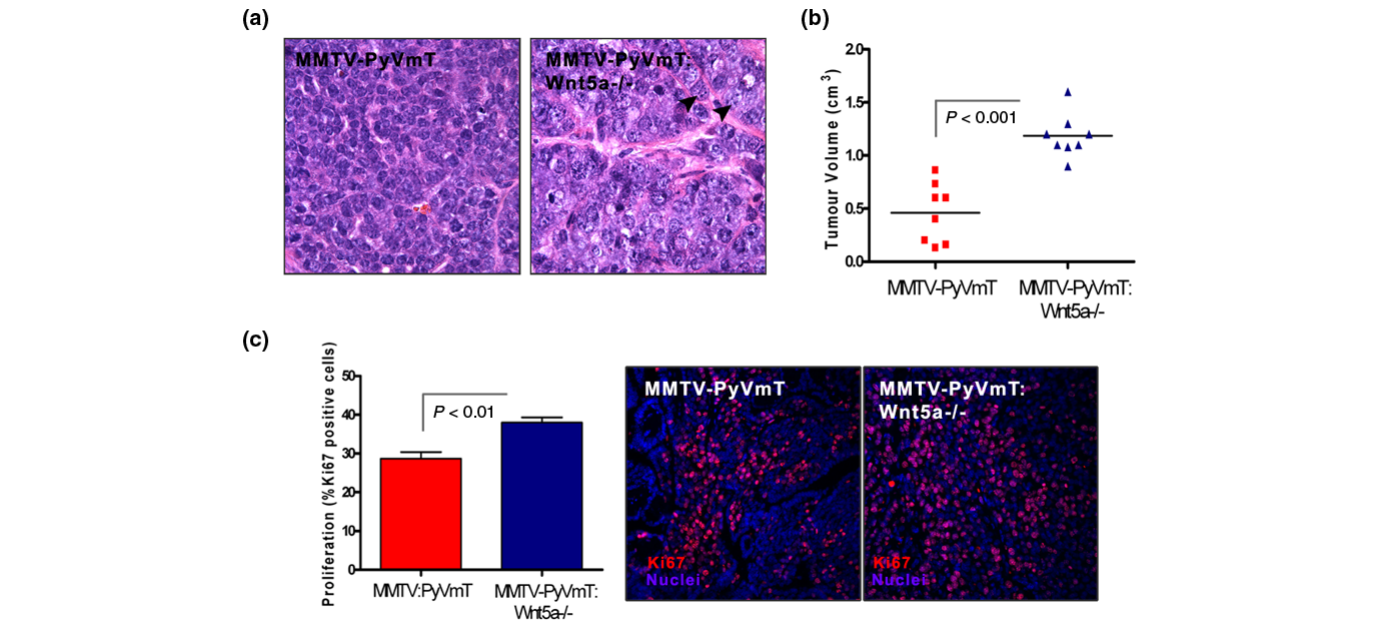
Absence of *Wnt5a *enhances growth of MMTV-PyVmT tumours. **(a) **Representative bright field images of H&E-stained MMTV-PyVmT and MMTV-PyVmT:Wnt5a-/- tumours. Arrows denote the large pleomorphic nuclei in *Wnt5a*-/- tumours. **(b) **Tumour volume at the time of sacrifice. Tumour volume is significantly larger in the absence of *Wnt5a *(eight per group, PyVmT mean tumour volume 0.46 cm^3^, PyVmT:Wnt5a-/- mean tumour volume 1.1 cm^3^; *P *< 0.0001). **(c) **Proliferation of MMTV-PyVmT:Wnt5a-/- and control MMTV-PyVmT tumours. Ki67 staining illustrates a significant increase in proliferation in the absence of *Wnt5a *(*P *< 0.0001, eight MMTV-PyVmT:Wnt5a-/- sections, three fields per section; four MMTV:PyVmT sections, three fields per section). DNIIR = dominant-negative transforming growth factor-β type II receptor; H&E = haematoxylin and eosin; MMTV = mouse mammary tumour virus promoter/enhancer; PyVmT = polyoma virus middle T antigen; Wnt = Wingless-related protein family.

To address the role of Wnt5a in mammary tumour formation, we derived MMTV-PyVmT and MMTV-PyVmT: *Wnt5a*-/- epithelium by rescue of embryonic mammary anlagen as previously described [5]. After 16 weeks, tumours in each group were classified as invasive adenocarcinomas. Histological evaluation of the tumours by H&E staining revealed the presence of pleomorphic nuclei with high nuclear to cytoplasmic ratios in MMTV-PyVmT:Wnt5a-/- tumours when compared with control MMTV-PyVmT tumours (Figure 2a). In the absence of *Wnt5a*, tumour volume was approximately two-fold greater than control MMTV-PyVmT tumours at the time of sacrifice (Figure 2b). By Ki67 staining, PyVmT:Wnt5a-/- epithelium exhibited a significant increase in proliferation compared with controls (Figure 2c). Together, the data demonstrate that alterations in TGF-β signalling or loss of *Wnt5a *enhance tumour growth.

**Figure 3:**
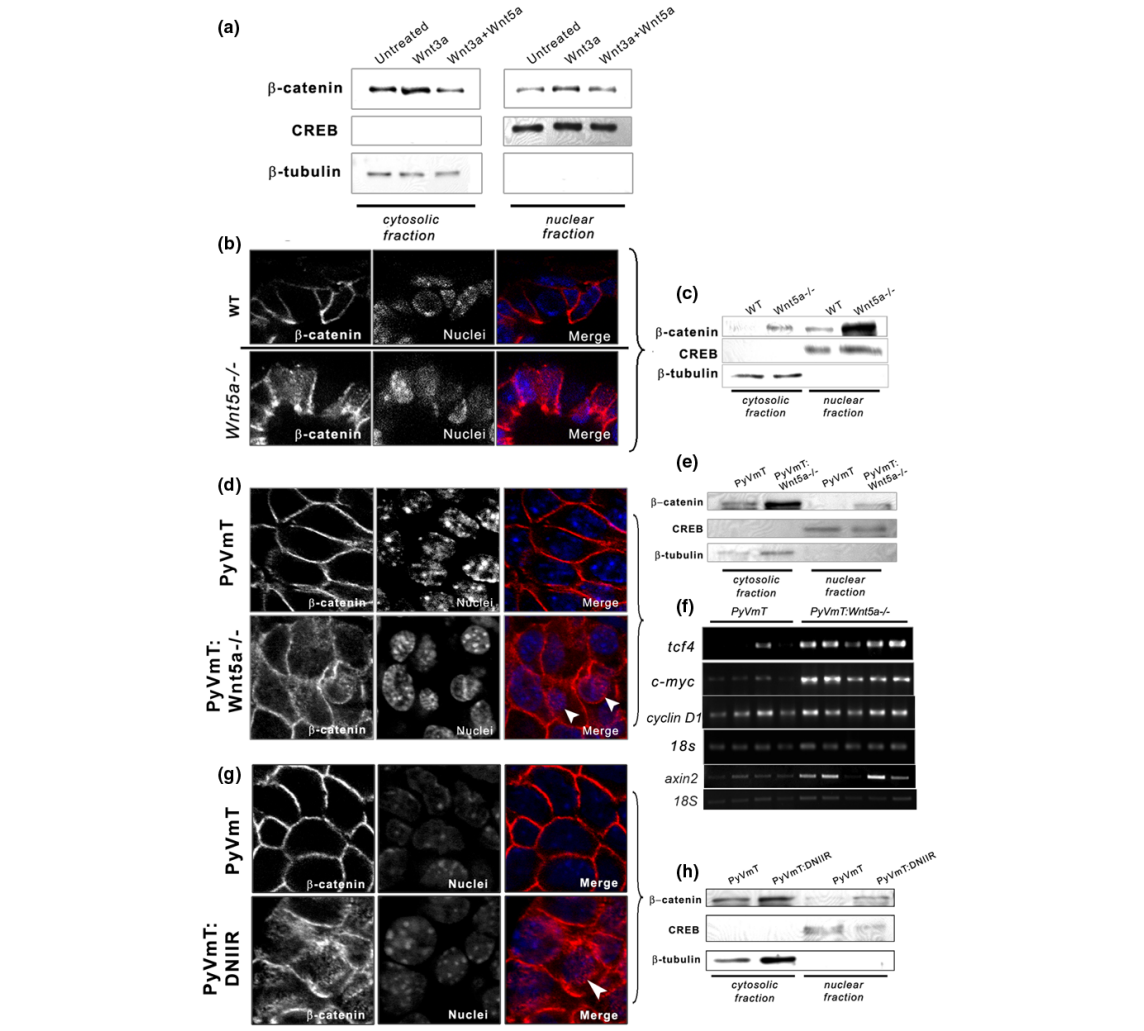
TGF-β and Wnt5a can inhibit the Wnt/β-catenin pathway. **(a) **Western blot of β-catenin protein from fractionated primary mammary epithelial cells after overnight stimulation with 60 ng/ml rWnt3a alone or in combination with 80 ng/ml rWnt5a. rWnt3a elevated nuclear β-catenin levels in epithelial cells, while rWnt5a inhibited rWnt3a-induced levels. **(b) **Immunofluorescent staining and confocal imaging for β-catenin in sections of WT and *Wnt5a*-/- mammary epithelium after three weeks of development illustrating nuclear and cytoplasmic localisation of β-catenin in *Wnt5a*-/- epithelium. A representative of five separate glands is shown. **(c) **Western blot shows the accumulation of β-catenin in the nuclear compartment of *Wnt5a*-/- epithelium relative to WT epithelium. **(d) **β-catenin localisation in MMTV-PyVmT and MMTV-PyVmT:Wnt5a-/- tumours (five tumours per group). The arrows point to examples of nuclei. **(e) **Subcellular fractionation and western blotting for β-catenin protein show elevated β-catenin levels in the nucleus of cells from *Wnt5a*-/- tumours. **(f) **Semi-quantitative RT-PCR using cDNA generated from individual MMTV-PyVmT and MMTV-PyVmT:Wnt5a-/- tumours, exhibiting increased Wnt/β-catenin transcriptional responses in the absence of *Wnt5a *(four control tumours, five *Wnt5a*-/- tumours). 18S was used to normalise for the amount of total RNA. PCR product is shown in the linear range (25 cycles for axin, tcf4, cyclin D1 and c-myc, and 15 cycles for 18S). **(g) **Immunofluorescent staining and confocal imaging (representative of four tumours per group), and **(h) **representative western blot of proteins from fractionated tumours, together, illustrating an increase in intracellular β-catenin when TGF-β signalling is blocked via overexpression of DNIIR. Arrows point to examples of nuclei. CREB is used as a loading control for the nuclear fraction, β-Tubulin is used as a loading control for the cytoplasmic fraction. DNIIR = dominant-negative TGF-β type II receptor; H&E = haematoxylin and eosin; MMTV = mouse mammary tumour virus promoter/enhancer; PyVmT = polyoma virus middle T antigen; RT-PCR = reverse transcripase polymerase chain reaction; TGF-β = transforming growth factor-beta; Wnt = Wingless-related protein family; WT = wild type, CREB+ c-AMO response element binding factor.

Since others have observed high nuclear to cytoplasmic ratios and pleomorphic nuclei associated with Wnt1-induced tumours [24] and it has been suggested that Wnt5a can antagonise the Wnt/β-catenin pathway [7-10], we tested the hypothesis that Wnt5a is a negative regulator of the Wnt/β-catenin pathway in the mammary gland. To determine if Wnt5a could antagonise the Wnt/β-catenin pathway in mammary epithelium, primary cells in culture were treated with a recombinant canonical signalling Wnt, rWnt3a, alone or with rWnt3a and rWnt5a in combination. After overnight treatments, rWnt3a increased the levels of nuclear β-catenin, while rWnt5a was able to suppress rWnt3a-induced nuclear β-catenin levels (Figure 3a). The results suggested that primary mammary epithelium is capable of responding to canonical Wnt and that Wnt5a can antagonise this response.

Next, we investigated β-catenin levels in *Wnt5a*-/- and wild type (WT) mammary epithelium, three weeks post-transplantation. During adolescence, TOPGAL (Wnt responsive β-galactosidase activity) activity, a measure of canonical Wnt/β-catenin signalling in the mammary gland, is very low to undetectable [25], so we reasoned that if there were changes in intracellular β-catenin due to loss of *Wnt5a*, they should be detectable at this stage. Little to no β-catenin was detected by confocal microscopy in the cytoplasm or nucleus of sections from WT glands. Interestingly, *Wnt5a*-/- epithelium exhibited increased levels of intracellular β-catenin compared with WT (Figure 3b). Furthermore, subcellular fractionation and protein isolation from WT and *Wnt5a*-/- epithelium followed by western blot analysis demonstrated an increase in the accumulation of nuclear β-catenin in the absence of *Wnt5a *(Figure 3c). The results suggested that Wnt5a normally acts to inhibit the stabilisation β-catenin in the developing mammary gland.

To determine if loss of *Wnt5a *also affected stabilisation of β-catenin in MMTV-PyVmT tumours, we analysed MMTV-PyVmT and MMTV-PyVmT:Wnt5a-/- tumours for differences in β-catenin levels. Immunostaining and confocal microscopy on sections from control and *Wnt5a*-/- tumours revealed elevated levels of intracellular β-catenin in the absence of *Wnt5a *(Figure 3d). Control MMTV-PyVmT tumours exhibited predominantly membrane-associated β-catenin. Moreover, subcellular fractionation and western blotting showed an increase in β-catenin in the nuclear compartment of cells in the *Wnt5a*-/- tumours (Figure 3e). Analysis of Wnt/β-catenin target genes such as c-myc [26], tfc4 [27] and axin 2 (Figure 3f) by RT-PCR indicated that the transcriptional activity of the Wnt/β-catenin pathway was enhanced in the absence of *Wnt5a*. Cyclin D1 was only modestly regulated. Thus, the data suggest that loss of *Wnt5a *results in increased canonical Wnt/β-catenin signaling in MMTV-PyVmT tumours.

### Accumulation of intracellular β-catenin occurs in tumours with dominant-negative interference of TGF-β signalling

Upon determining that Wnt5a limits the accumulation of intracellular β-catenin in normal mammary epithelium and tumours, we speculated that tumours harboring DNIIR would also exhibit stabilised intracellular β-catenin, probably as a consequence of reduced Wnt5a levels. To confirm this, MMTV-PyVmT tumours with intact or altered TGF-β signalling were immunostained for β-catenin. The accumulation of intracellular of β-catenin was apparent by confocal microscopy in the DNIIR tumours relative to control tumours where the majority of β-catenin was membrane-associated (Figure 3g). This pattern was observed in MMTV-Neu;DNIIR tumours as well as in the developing DNIIR mammary gland (data not shown). Moreover, subcellular fractionation of the cells in PyVmT tumours revealed elevated levels of nuclear β-catenin in DNIIR tumours compared with the control group (Figure 3h). The results suggest that loss of responsiveness to TGF-β also results in an increase in Wnt/β-catenin signalling in mammary tumours.

### DNIIR and Wnt5a-null tissue demonstrate an increase in Sca1, K6 and K14 expression

**Figure 4:**
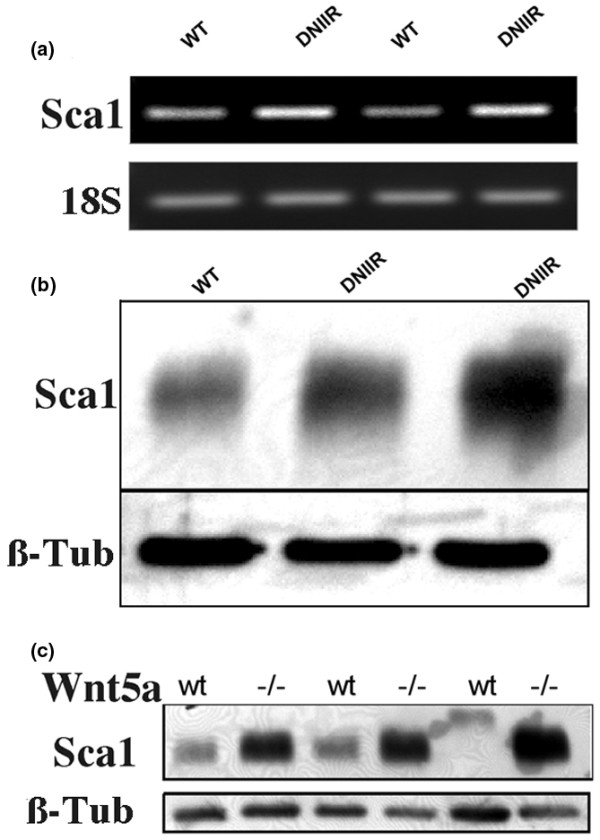
Sca1, a marker of mammary stem/progenitor cells, is increased in DNIIR and *Wnt5a*-/- glands. mRNA and protein were extracted from several wild type (WT) and MT-DNIIR (DNIIR) mice. **(a) **Sca1 mRNA levels were determined by semi-quantitative RT-PCR and **(b) **protein levels were determined by western blot. Sca1 levels were increased in DNIIR glands relative to controls. **(c) **Sca1 protein levels were determined in WT and *Wnt5a *null (-/-) glands using western blot analysis. Sca1 levels were increased in *Wnt5a*-null tissue relative to controls. 18S and β-Tubulin (β-Tub) were used as normalisation controls. DNIIR = dominant-negative transforming growth factor-β type II receptor; RT-PCR = reverse transcripase polymerase chain reaction; Wnt = Wingless-related protein family.

We originally identified Wnt5a as a target of TGF-β action in a microarray screen comparing WT and DNIIR mammary glands [5]. In addition to alterations in expression of Wnt5a, up-regulation of Sca1 was detected in this original microarray screen (data not shown). Interestingly, MMTV-Wnt1 mice also demonstrate increased Sca1 expression in the mammary gland [28,29]. To confirm the results seen in the microarray, regulation of Sca1 mRNA was compared in WT and DNIIR glands using RT-PCR (Figure 4a). Sca1 mRNA was clearly up-regulated in the mammary glands of two separate DNIIR mice when compared with controls. To determine if Sca1 protein was also up-regulated, we used western blot analysis (Figure 4b). The levels of Sca1 protein were increased in DNIIR glands compared with controls.

To determine if loss of *Wnt5a *also resulted in an increase in Sca1 expression, we compared Sca1 protein levels in tissue rescued from three separate WT and *Wnt5a*-/- mice by western blot (Figure 4c). Loss of *Wnt5a *also resulted in a significant increase in Sca1 protein levels in the mammary gland relative to WT controls.

**Figure 5:**
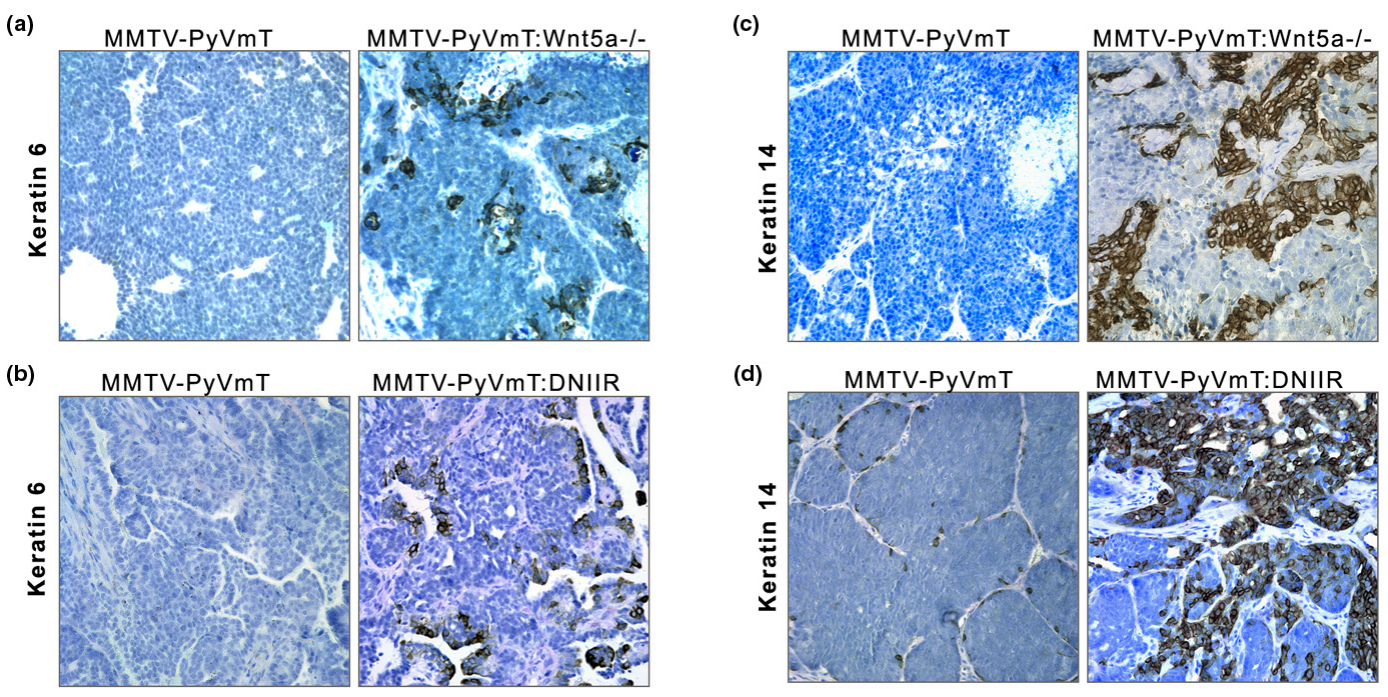
K6 and K14 expressing populations are increased in Wnt5a-/- and DNIIR tumours. Immunohistochemistry for K6 demonstrating the expansion of this progenitor population in **(a) **MMTV-PyVmT:Wnt5a-/- and **(b) **MMTV-PyVmT:DNIIR tumours compared with control groups (five per group). Immunohistochemistry for K14 in **(c) **MMTV-PyVmT:Wnt5a-/- and **(d) **MMTV-PyVmT:DNIIR tumours, along with representative controls (five per group). The K14 population in both Wnt5a-/- and DNIIR tumours displays a unique rounded morphology. DNIIR = dominant-negative transforming growth factor-β type II receptor; K = keratin; MMTV = mouse mammary tumour virus promoter/enhancer; PyVmT = polyoma virus middle T antigen; Wnt = Wingless-related protein family.

We next tested the hypothesis that the absence of *Wnt5a *or TGF-β signalling would redirect the phenotype of MMTV-PyVmT tumours to a phenotype consistent with activated Wnt/β-catenin signalling. A previous study demonstrated the expansion of a K6 expressing population of cells in tumours that are induced by components of the Wnt/β-catenin signalling pathway, including Wnt1, c-Myc and β-catenin, yet not in tumours induced by other oncogenes such as neu, PyVmT and Ras [29]. Although this K6 population was not observed in MMTV-PyVmT tumours, we speculated that because MMTV-PyVmT:Wnt5a-/- tumours display higher levels of intracellular β-catenin and *Wnt5a*-/- tissue demonstrates increased expression of Sca1, an expansion of this K6-positive population might exist. Using immunohistochemistry we detected the presence of a K6 population of cells in PyVmT tumours in the absence of *Wnt5a *(Figure 5a), suggesting an expansion of this population in the tumours. Interestingly, PyVmT tumours expressing DNIIR also displayed an increase in the K6 expressing population (Figure 5b).

Li and colleagues [29] also established the existence of an expanded myoepithelial lineage in tumours induced by excessive canonical Wnt signalling. The expansion of this myoepithelial lineage was not observed in tumours transformed by oncogenes such as PyVmT. MMTV-PyVmT tumours, in the absence of *Wnt5a *also contained an expanded myoepithelial lineage, marked by K14 (Figure 5c). The myoepithelial lineage seemed to mimic what was observed in Wnt/β-catenin-induced tumours, with enlarged and rounded morphology [29]. MMTV-PyVmT:DNIIR tumours also contained this unusual K14 positive population (Figure 5d). These results suggest that alterations in TGF-β signalling and deletion of *Wnt5a *promote the expansion of a myoepithelial lineage typically observed in canonical Wnt-induced tumours. Together the results suggest that loss of TGF-β or Wnt5a can redirect the phenotype of MMTVPyVmT tumours to a phenotype consistent with activated Wnt/β-catenin.

## Discussion

In this study, we present evidence to support a model in which TGF-β and a non-canonical signalling Wnt, Wnt5a, act as antagonists of the Wnt/β-catenin pathway in the mammary gland. Furthermore, DNIIR and *Wnt5a*-/- tumours show similarities to tumours induced by activation of Wnt/β-catenin in their ability to expand a K6 progenitor cell population in addition to a unique K14 expressing population. Specifically, we propose that TGF-β inhibits Wnt/β-catenin signalling through the regulation of Wnt5a expression and that alteration in TGF-β signalling via dominant-negative interference or Wnt5a signalling, using *Wnt5a*-/- mammary tissue, promote the stability of intracellular β-catenin and redirect the formation of tumours to a Wnt/β-catenin phenotype.

Several lines of evidence support a model in which TGF-β signalling has opposing effects on Wnt/β-catenin signalling in the mammary gland. Transgenic mice that overexpress a constitutively active form of β-catenin, ΔN89β, under the MMTV promoter/enhancer display precocious lobuloalveolar development and differentiation in the mammary glands of virgin mice [30]. Conversely, overexpression of Axin, a negative regulator of β-catenin, results in defects in lobuloalveolar development during pregnancy [31]. This is similar to studies demonstrating that interference of TGF-β signalling during mammary development induces precocious alveolar differentiation in mice, while targeted overexpression of active TGF-β1 inhibits alveolar development and lactation [2].

In addition to the mammary gland phenotypes, downstream signalling targets point toward opposing roles for TGF-β and Wnt/β-catenin pathways. Proteins shown to be upregulated by the Wnt/β-catenin pathway, such as c-myc, cyclin-D1 and Id, are usually downregulated by the TGF-β pathway [32-34]. Moreover, studies in other model systems provide direct evidence that the TGF-β pathway can suppress the Wnt/β-catenin pathway [35-37]. For example, bone morphogenic protein-2 (BMP-2), a TGF-β family member, can inhibit Wnt signalling in osteoblast progenitors via a Smad1/Dvl interaction [38] and, additionally, TGF-β signalling can dictate the size of the dorsal midbrain by antagonising canonical Wnt signalling and negatively regulating self-renewal of neuroepithelial stem cells [36]. Together with results presented here there is strong evidence for antagonistic interactions between TGF-β and Wnt/β-catenin pathways in the mammary gland.

Despite significant evidence in favour of antagonism between TGF-β and Wnt/β-catenin signalling, other studies suggest that these two pathways act together [39]. The extent of cooperation ranges from similar mutant phenotypes between β-catenin and Smad2/4 during gastrulation, the necessity of TGF-β/Smad in mediating β-catenin responses such as epithelial mesenchymal transition and apoptosis, and also the presence of LEF1 and Smad binding sites in certain promoters. Moreover, a recent study demonstrated a unique TGF-β/Wnt responsive gene signature in mammary and intestinal cancers [39]. Given the complexity and biphasic nature of TGF-β in mammary tumourigenesis, it is likely that our hypothesis that TGF-β acts to inhibit Wnt/β-catenin signalling, and the hypotheses of others that suggest cooperation between TGF-β and Wnt signalling are not mutually exclusive. Our studies propose that alterations in TGF-β signalling promote the stabilisation of intracellular β-catenin, enhancing mammary tumourigenesis, but the effects of TGF-β on late-stage tumour progression, such as those associated with epithelial mesenchymal transition and metastasis, might involve cooperation with Wnt signalling.

The heterogeneity of breast cancers has sparked interest in the presence of a population of cells termed 'cancer stem cells' or 'tumour-initiating cells' that are small in number, undifferentiated and can give rise to differentiated lineages that form the bulk of the tumour [40]. The specific initiating oncogenic pathway present in genetically engineered mouse models is thought to create distinct pathological features [41,42]. Along with the oncogenic stimulus, studies have demonstrated that the cell of origin also can dictate pathological features observed in tumours. Although MMTV-PyVmT is thought to transform a more differentiated cell-type in the mammary gland [23], it has been proposed that activation of Wnt/β-catenin signalling supports the expansion of a progenitor population in tumours [29,41,42]. In our model, we demonstrated that in the context of MMTV-PyVmT, a K6 progenitor population and an unusual K14 expressing population were expanded in the absence of *Wnt5a *or when TGF-β signalling was interrupted. The results suggest that TGF-β and Wnt5a redirect tumour phenotype to form tumours similar to those formed by excess Wnt/β-catenin signalling. By extension, it is reasonable to propose that TGF-β and Wnt5a may also regulate the stem cell population.

The existence of self-renewing, multipotent cells in the developing mammary gland has been known for many years [18]. Fragments of mouse mammary epithelium transplanted into a cleared fat pad can regenerate the entire functional gland. Subsequent serial transplantation and regeneration of the initial outgrowths demonstrated the existence of the self-renewing population. Later studies showed that this stem cell population was enriched for Sca1, which was originally identified as a marker of haematopoietic cells [28]. It has been suggested that one of the major roles of canonical Wnts in the skin, intestine and other tissues including the breast is to regulate maintenance and proliferation of stem cells [43,44]. TGF-β, however, can limit the stem cell population and facilitates the differentiation of this population to a more committed state [45-47]. We propose a model in which TGF-β acting through Wnt5a limits Wnt/β-catenin signalling and the stem cell population. Due to controversies regarding stem cell markers, testing this model will require extensive cell profiling and functional studies to re-populate cleared fat pads.

In summary, the similarities observed in *Wnt5a*-/- tumours to those tumours derived from components of the Wnt/β-catenin pathway provide the first evidence that Wnt5a acts to inhibit the Wnt/β-catenin pathway in the mammary gland. The fact that Wnt5a is regulated by TGF-β provides another interesting facet that warrants further investigation with respect how these two factors are linked throughout mammary gland development, in the regulation of stem cell dynamics and during mammary tumourigenesis.

## Conclusions

Previously, we showed that Wnt5a is a direct target of TGF-β signalling and is required for many of the effects of TGF-β in mammary development. The key findings here are that TGF-β and Wnt5a antagonise Wnt/β-catenin signalling in mammary tissue and that loss of TGF-β or Wnt5a signalling can redirect the phenotype of mammary tumours to that resembling tumours induced by excessive Wnt/β-catenin. These findings provide a new mechanism whereby TGF-β could act as a tumour suppressor.

## Abbreviations

BMP-2: bone morphogenic protein-2; BSA: bovine serum albumin; DMBA: 7, 12-dimethybenz(a)anthracene; DMEM: Dulbecco's modified eagle's medium; DNIIR: dominant-negative TGF-β type II receptor; EGF: epidermal growth factor; FBS: fetal bovine serum; HBSS: Hanks' balanced salt solution; HEPES: 4-(2-hydroxyethyl)-1-piperazineethanesulfonic acid; H&E: haematoxylin and eosin; K: keratin; MMTV: mouse mammary tumour virus promoter/enhancer; PBS: phosphate-buffered saline; PyVmT: polyoma virus middle T antigen; RT-PCR: reverse transcripase polymerase chain reaction; TGF-β: transforming growth factor-beta; TGF-βRI: type I TGF-β receptor; TGF-βRII: type II TGF-β receptor; Wnt: Wingless-related protein family; WT: wild type.

## Competing interests

The authors declare that they have no competing interests.

## Authors' contributions

KR carried out the PyVmT studies and did the analysis and staining of the tumour tissue. He also drafted the paper. SEB performed some of the RT-PCR experiments as well as the Sca1 experiments. MRC set up the MMTV-neu study and performed the original microarray experiments. ARF helped to analyse the data by classifying tumours and reading the immunostaining on the tumour samples. RS participated in the design and coordination of the study as well as interpretation of the data. RS also edited the final manuscript.
